# Do disease status and race affect the efficacy of zoledronic acid in patients with prostate cancer? A systematic review and meta-analysis of randomized control trials

**DOI:** 10.1371/journal.pone.0275176

**Published:** 2022-09-22

**Authors:** Chiwei Chen, Mandi Lin, Daocheng Yu, Weiting Qin, Jianfu Zhou, Lang Guo, Renlun Huang, Xinxiang Fan, Songtao Xiang

**Affiliations:** 1 Department of Urology, The Second Affiliated Hospital of Guangzhou University of Chinese Medicine, Guangzhou, Guangdong, China; 2 Department of Radiotherapy, The First Affiliated Hospital of Guangzhou University of Chinese Medicine, Guangzhou, Guangdong, China; 3 Department of Urology, The First Affiliated Hospital of Guangzhou University of Chinese Medicine, Guangzhou, Guangdong, China; 4 Department of Urology, Sun Yat-sen Memorial Hospital of Sun Yat-sen University, Guangzhou, Guangdong, China; Hospital de Santa Maria, PORTUGAL

## Abstract

**Background:**

Zoledronic acid (ZA) does not improve the overall survival (OS) of metastatic castration-resistant prostate cancer (mCRPC); however, little is known about the efficacy of ZA in to hormone-sensitive prostate cancer (HSPC), metastatic hormone-sensitive prostate cancer (mHSPC), and non- metastatic castration-resistant prostate cancer (nmCRPC). Therefore, we assessed the efficacy of ZA in patients with prostate cancer (PCa) and different disease statuses.

**Methods:**

Fifteen eligible randomized-control trials (RCTs) with ZA intervention, including 8280 participants with HSPC, mHSPC, nmCRPC, and mCRPC, were analyzed. The primary and secondary outcome were overall survival(OS), and skeletal-related events (SREs), and bone mineral density (BMD).

**Results:**

The participants included 8280 men (7856 non-Asian and 424 Asian). Seven trials yielded a pooled hazard ratio (HR) of 0.95 (0.88, 1.03; *P* = 0.19) for OS. Subgroup analysis revealed no significant improvement in OS in the HSPC, castration-resistant prostate cancer (CRPC), M0 and M1(bone metastasis) groups, with pooled HR (95%CI) of 0.96 (0.88,1.05), 0.78 (0.46,1.33), 0.95 (0.81,1.13), 0.85 (0.69,1.04) respectively. The Asian group exhibited improved in OS with an HR of 0.67 (0.48, 0.95; *P* = 0.02), whereas the non-Asian group showed no improvement in OS with an HR of 0.97 (0.90, 1.06; *P* = 0.52). Five trials yielded pooled odds ratio (OR) of 0.65 (0.45, 0.95; *P* = 0.02) for SREs. In the subgroup, SREs were significantly decreased in the M1 and Asian groups with ORs of 0.65 (0.45, 0.95; *P* = 0.02) and 0.42 (0.24, 0.71; *P* = 0.001), respectively. Six trials yielded a pooled mean difference (MD) of 8.08 (5.79, 10.37; P < 0.001) for BMD. In the HSPC we observed a stable improvement in increased BMD percentage with an MD (95%CI) of 6.65 (5.67, 7.62) (*P* = 0.001).

**Conclusions:**

ZA intervention does not significantly improve OS in patients with prostate cancer (HSPC, CRPC, M0, M1) but probably improves OS in the Asian populations. M1 and Asian groups had exhibit a significant reduction in SREs regardless of the HSPC or CRPC status after ZA administration. Moreover, ZA treatment increases BMD percentage.

## Introduction

Recently published data suggested that approximately 1.6 million men are diagnosed PCa, which causes approximately 366, 000 deaths each year [1]. Owing to the frequency of bone metastasis in PCa and its high cancer-specific mortality, research on bone metastasis is urgent and important.

ZA is the most potent bisphosphonates and is currently recommended for the management of bone metastasis in various solid tumors. It functions as an adjunctive treatment and bone-targeted therapy for supportive care with mCRPC [2]. In recent years, many studies have focused on the effects of ZA on OS. A randomized-controlled trial (RCT) demonstrated that there was no OS improvement compared to the non-ZA group in patients with mCRPC [3], whereas another RCT reported a contrary outcome [4]. Notably, basic and preclinical experiments revealed that ZA could exert an anti-tumor effect in *vivo* and in *vitro* in PCa, breast cancer, cervical cancer, and osteosarcoma [5–8]. However, little is known about the effect of ZA on OS of other PCa stages such HSPC, mHSPC), and nmCRPC.

SREs were established as primary endpoints in the evaluation of ZA efficacy which was proven to reduce SREs in patients with breast cancer bone metastasis and CRPC bone metastasis. The guidelines also recommended administering ZA to patients with mCRPC [9]. Unfortunately, its effects on nmCRPC and HSPC remain unclear. Only a few studies have revealed weak evidence for the efficacy of ZA in mHSPC [10,11]. ZA can prevent bone loss, which reflects the BMD of HSPC [12], however, its effect on SREs in the HSPC stage remains unclear. Therefore, exploring the optimal efficacy of ZA is crucial.

Evidence of ZA intervention in different PCa statuses and outcomes must be obtained. Therefore, we performed a systematic review and meta-analysis to identify the ZA’s contradictory efficacy and to explore its role in the disease and metastatic status of PCa.

## Methods

This review was performed according to the preferred reporting criteria for systematic reviews and meta-analysis. We adhered to the Preferred Reporting Items for Systematic Reviews and Meta-Analyses (PRISMA) guidelines. The protocol is registered in the PROSPERO register (CRD42020223634).

### Search strategy

We followed the recommendations of a meta-analysis of RCTs in the epidemiological group to perform this search strategy. Four databases were searched: PubMed, Embase, the Cochrane Library, and the China National Knowledge Infrastructure (CNKI) on December 31, 2020. The search strategy was implemented using combined index terms (Medical Subject Headings, Emtree) and free-text keywords. Keywords included (“prostate cancer” OR “prostate neoplasm”) AND (“Zoledronic acid” OR “2-(lmidazol-1-yl)-1-hydroxyethylidene-1” OR “1-bisphosphonic”) AND (“Randomized Controlled Trial”). Other trial sources were searched for by examining the reference lists of the reviews, ongoing trials, and publications eligible for potential trials.

### Trials selection and data extraction

**Fig 1:**
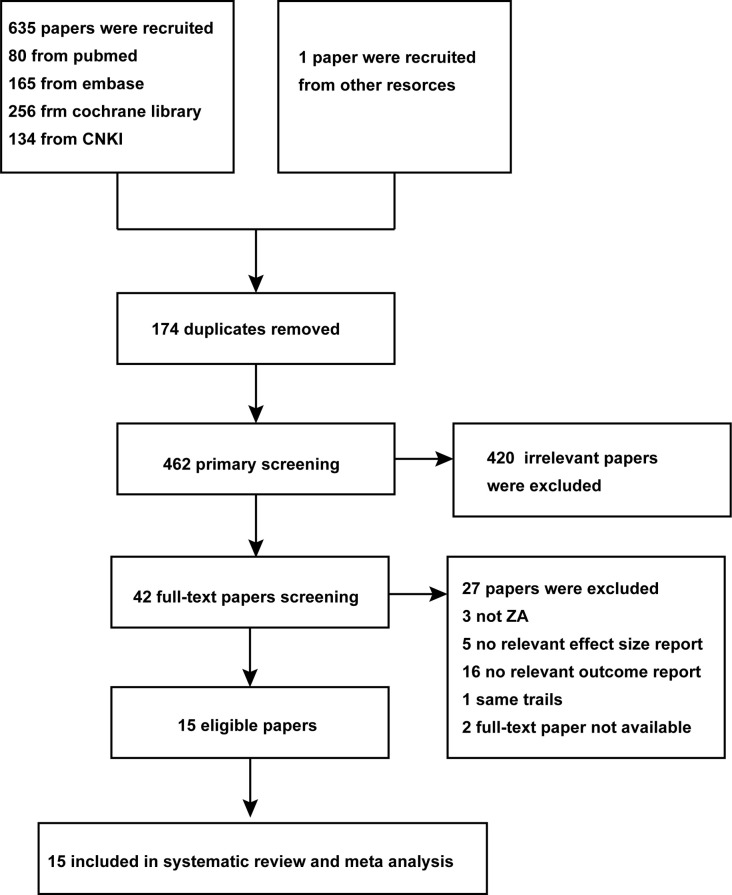
Flow chart to screen eligible studies.

The study participants included patients at all stages of PCa who did not undergo radical prostatectomy. We evaluated OS as the primary outcome and SREs and bone mineral density (BMD) as the secondary outcomes. Trials were eligible if they were randomized controlled trials; (2) investigated patients with PCa who were treated with ZA; (3) assessed OS, SREs, or BMD; and (4) provided HRs or odds ratios (ORs), and their 95% confidence intervals (CIs), SREs, and control events with means and SD of increased BMD percentage. Studies were excluded if they (1) had participants with malignancies other than PCa, and (2) if duplicate articles were derived from an identical or overlapping patient population, only the latest and/or complete article was used in the meta-analysis (Fig 1).

Two reviewers independently selected the trials for inclusion and exclusion. If the two reviewers disagreed, a third reviewer screened the full text and gave an objective judgment based on evidence from the trial. For all eligible trials, the following data were collected: author, publication year, study name, country group, total number of participants included in the study, treatment group intervention, control, metastatic status, disease status, median age, Gleason score (GS), and median follow-up. For the meta-analysis, the HRs and their 95% CIs of OS, SREs, and control events, means, and SD of BMD percentage increased values were collected. The exact definitions of SREs varied among clinical trials [9]. In our study, SREs were defined as fracture, spinal cord compression, need for radiation therapy or surgery [13], and pain [14]. BMD of lumbar vertebrae L2–L4, which was detected by dual-energy X-ray absorptiometry, was also extracted. Additionally, we extracted the methods of sequence generation, allocation concealment, completeness of outcome data reporting, and attrition from trial reports and/or protocols (if possible, as described above) to assess the risk of bias in individual trials [15]. The Q test was performed to detect heterogeneity before using the fixed-effects or random-effects models. If I^2 ≥^ 50%, which was considered statistically significant for heterogeneity [16], we used random-effects models; otherwise, we used fixed-effects models [17]. Funnel plots were used to detect potential publication bias. Since fewer than 10 studies reported SRE outcomes and the BMD percentage increased value, a funnel plot was mapped only for studies that reported OS to evaluate publication bias. A leave-one-out sensitivity analysis was performed to assess the stability of the results. Meta-analysis was performed using the Review Manager software. Subgroup definitions were as follows: HSPC, CRPC, [18] M0 indicated no bone metastasis, whereas M1 indicated bone metastasis. Notably, race was challenging to define owing to the lack of race-associated data in each study. Thus, in this study, the race was defined as patients recruited in Asian countries; the subgroups of race and GS score were derived from their corresponding medians. Furthermore, two trials [19,20] studied different interventions (with or without docetaxel (DOC)) in the control group, and one trial [21] reported data based on a subgroup of pain or no pain that occurred in patients. Thus, we divided the different interventions (with or without DOC) and diverse symptoms (pain or no pain) into two subgroup studies to analyze this data

## Results

### Characteristics of eligible studies

**Fig 2:**
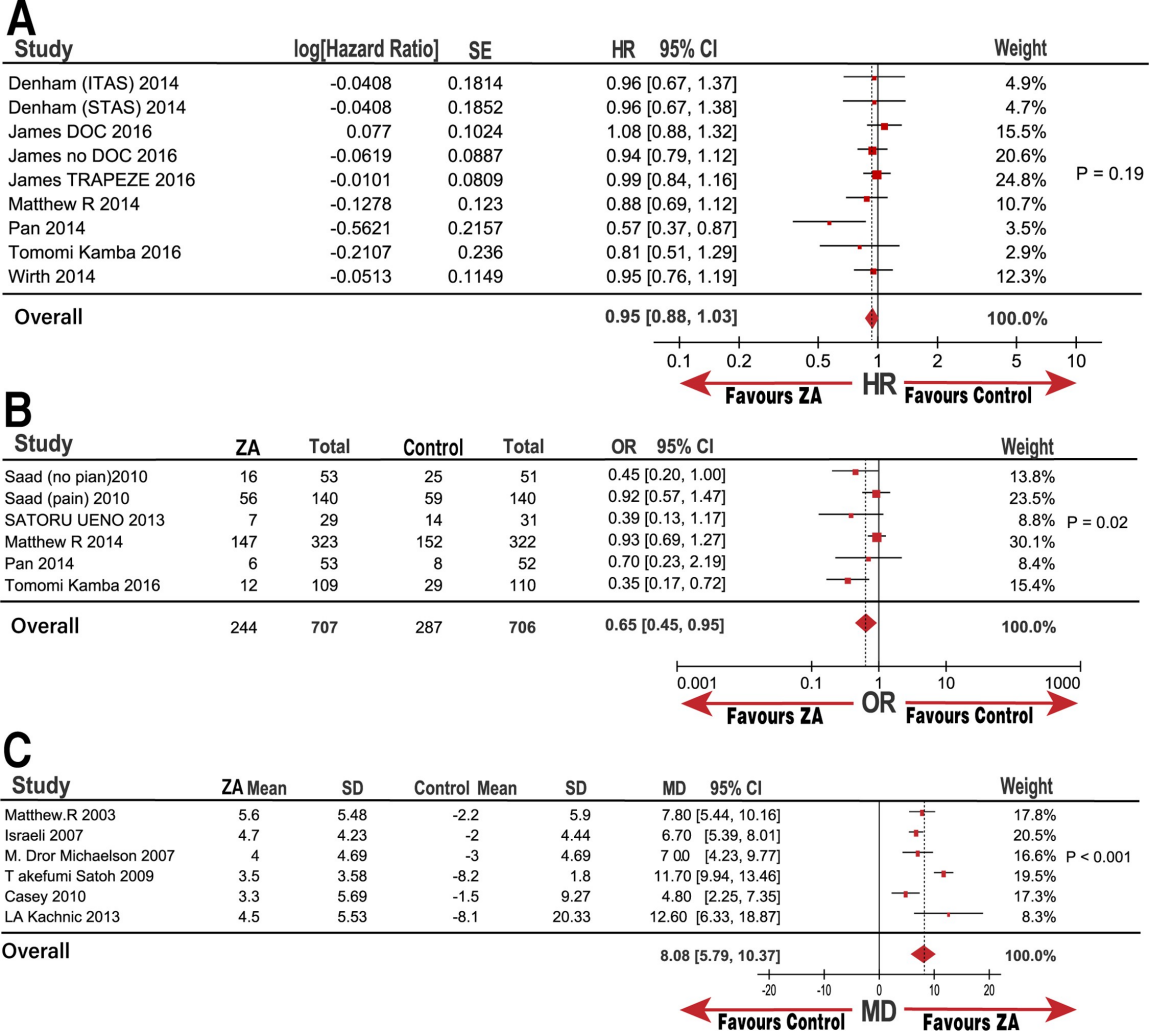
Forest plot with HR, OR, MD of OS comparing ZA with control group.

A total of 635 studies were reviewed, and 15 studies were extracted (Table 1) for the meta-analysis (Fig 2). A total of 8280 participants with PCa were enrolled from Asia, Europe, North America, and Oceania. Seven trials reported OS, five trials reported SREs, and six trials reported BMD. The details are listed in Table 1. Notably, we enrolled all PCa statuses in performing a complete ZA evaluation of every PCa status. The quality assessment of eligible studies is depicted in S1 Fig.

**Table 1:** Characteristics of trials.

Author	Year	trials name	Country	Number of patients	Treatment	Control	Metastatic status	Disease status	Median age	Gleason score of 8–10 (%)	Median follow-up (survival)
Nicholas D James [19]	2016	STAMPEDE (without DOC)	UK	1777	SOC+ZA	SOC	M0/M1	HSPC	66	69	43
Nicholas D James [19]	2016	STAMPEDE (with DOC)	UK	1185	SOC+DOC+ZA	SOC+DOC	M0/M1	HSPC	66	73	43
Nicholas D James [3]	2016	TRAPEZE	UK	757	DOC+ZA or DOC+ Sr89+ZA	DOC or DOC+ Sr89	M1	CRPC	68	-	22
Tomomi Kamba [22]	2016	ZAPCA	JAPAN	219	CAB+ZA	CAB	M1	HSPC	72	82.2	41.5
Yue Pan [4]	2014	/	CHINA	105	DOC+ Ca + VD+ ZA	DOC + Ca + VD	M1	CRPC	/	53	two years or more
Matthew R. Smith [23]	2014	CALGB 90202	USA	645	Androgen Deprivation Therapy +ZA	ADT+ placebo	M1	HSPC	66.3	58	11.8 months for the ADT+ZA 13.6 months for the ADT+ placebo
Manfred Wirth [24]	2014	ZEUS	EUROPE	1393	ADT+ZA	ADT	M0	HSPC	67	62.2	57.6
James W Denham [20]	2014	RADAR(STAS)	Australia and New Zealand	536	STAS+RT+ZA	STAS+RT	M0	HSPC	69	33	88.8
	2014	RADAR(ITAS)	Australia and New Zealand	535	ITAS+RT+ZA	ITAS+RT	M0	HSPC	68	39	88.8
SATORU UENO [14]	2013	ZABTON-PC	JAPAN	60	CAB+ZA	CAB	M1	HSPC	71.7	83.3	/
LA Kachnic [25]	2013	RTOG 0518	USA	96	ADT or RT+ VD+ Ca +ZA	ADT or RT +VD + Ca	M0	HSPC	70.5	68.8	36.3 months for ADT or RT+ZA and 34.8 months for ADT or RT
Rihard casey [26]	2010		CANADA	187	ADT + VD + Ca+ ZA	ADT + VD + Ca	M0	HSPC	/	/	12
Fred Saad [21] (Without pain)	2010		CANADA	104	SOC+ZA	SOC+ placebo	M1	CRPC	72.5	/	24
Fred Saad [21] (With pain)	2010		CANADA	280	SOC+ZA	SOC+ placebo	M1	CRPC	72.5	/	24
Takefumi Satoh [27]	2009		JAPAN	40	ADT+ZA	ADT+ placebo	M1	HSPC	70	/	12
Ron S. Israeli [28]	2007		USA	215	ADT+ZA	ADT+ placebo	M0	HSPC	73.5	/	/
M. Dror Michaelson [29]	2007		USA	40	ADT + VD + Ca+ ZA	ADT + VD + Ca	M0	HSPC	65.5	/	12
Matthew R. Smith [12]	2003		USA	106	ADT+ZA	ADT+ placebo	M0	HSPC	70.6	/	12

### Effect of ZA on the OS of patients with prostate cancer

The pooled data are shown in Fig 2. Seven trials (nine groups) yielded a pooled HR and 95% CIs of 0.95 (0.88, 1.03; P = 0.19) for OS based on the fixed-effects model that was used for analysis with a low heterogeneity (I^2^ = 4%, P = 0.4). The addition of ZA did not significantly improve the OS compared to that in the control group. No publication bias was observed by constructing a funnel plot (S2 Fig).

**Fig 3:**
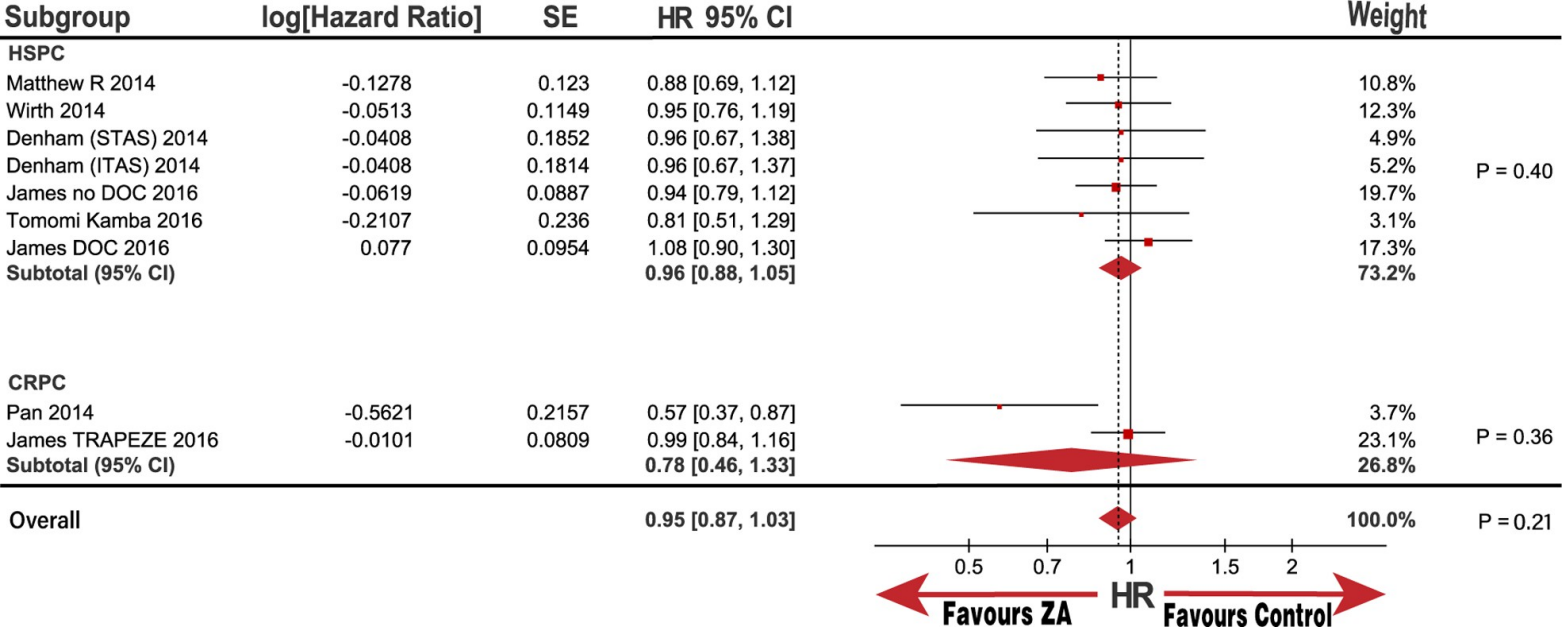
Forest plot with hazard ratio (HR) of OS in subgroup of HSPC and CRPC.

**Fig 4:**
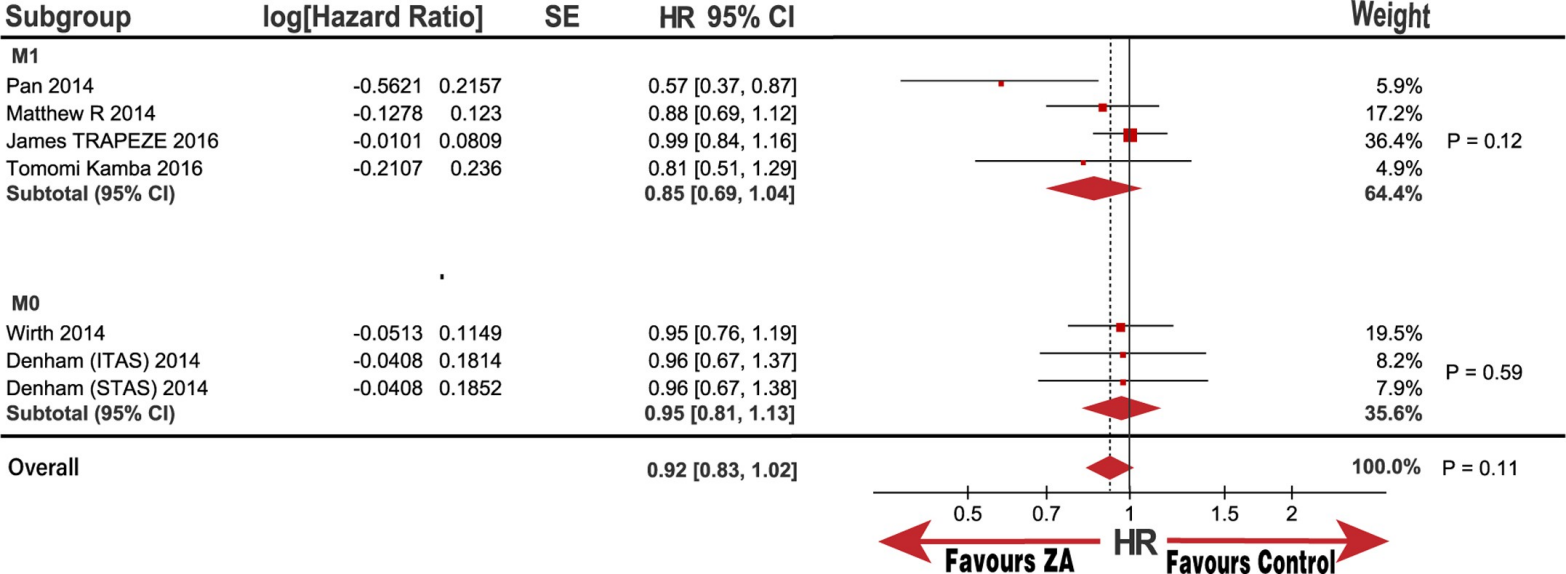
Forest plot with hazard ratio (HR) of OS in subgroup of M1 and M0.

**Fig 5:**
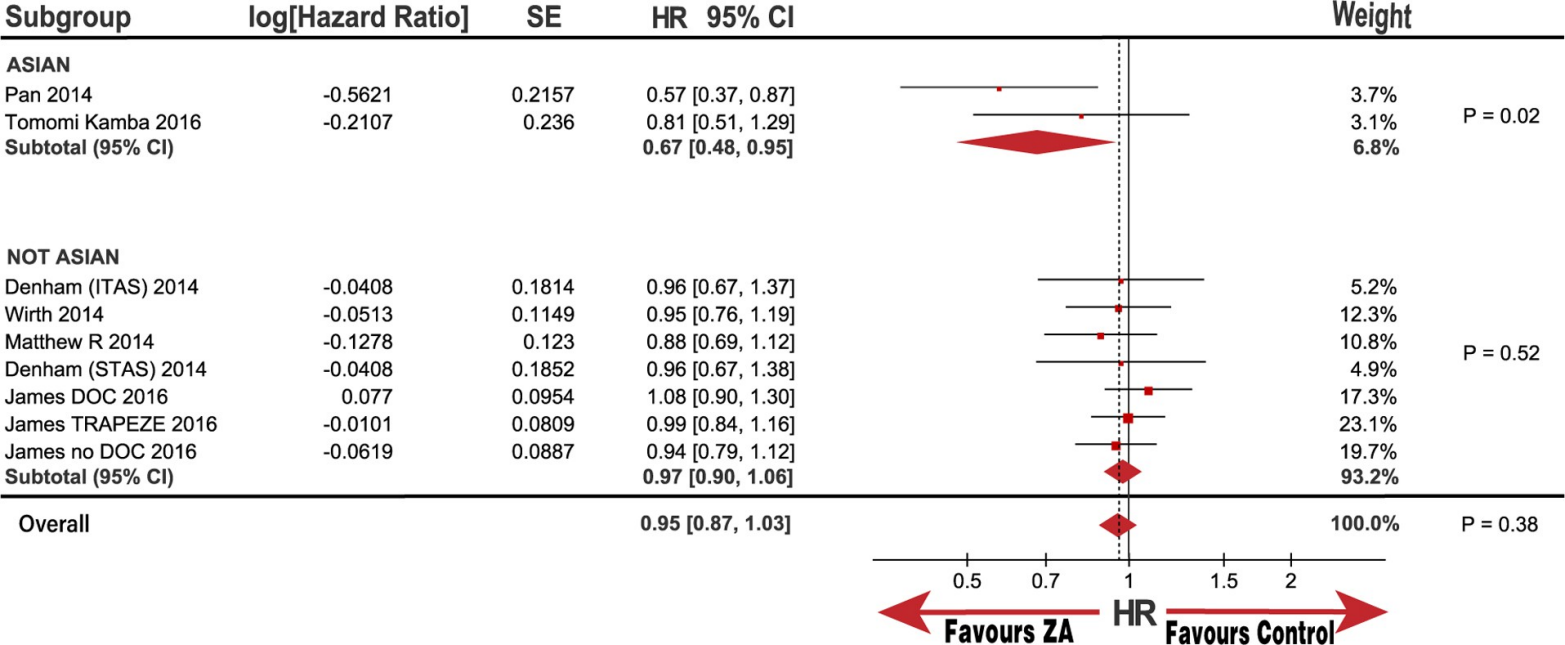
Forest plot with hazard ratio (HR) of OS in subgroup of Asian and not Asian.

**Fig 6:**
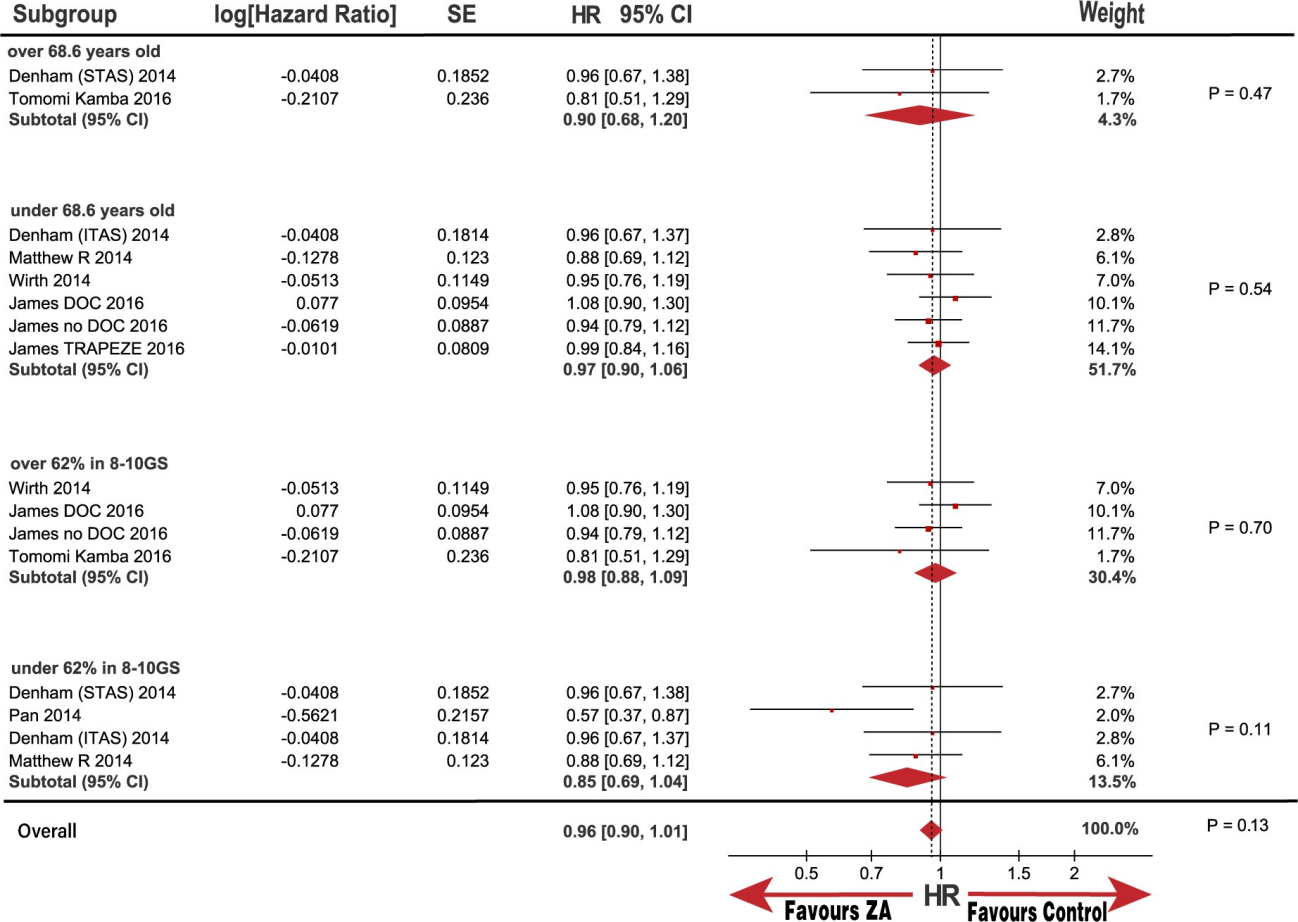
Forest plot with hazard ratio (HR) of OS in subgroup of age and GS.

OS subgroup analysis, categorized by disease status, metastatic status, interventions, and race, was also performed. Notably, the baseline race populations differed in age and patient proportion of 8 to 10 GS score (Table 2); therefore, we also performed subgroup analysis categorized by age and GS score. No significant improvement was observed in the OS in the HSPC subgroups or M0 metastasis with the pooled HRs (95% CI) of 0.96 (0.88, 1.05) and 0.95 (0.81, 1.13), respectively. Although there was no statistical significance in the CRPC and M1 metastasis subgroups, the heterogeneity was substantially high, with I^2^ values of 83% and 51%, respectively. (Figs 3 and 4). Notably, the outcome in the race subgroup suggested that the Asian group had a striking improvement in OS compared with that of the control group, with an HR (95% CI) of 0.67 (0.48, 0.95; P = 0.02). In contrast, the non-Asian group exhibited no improvement in OS compared with the control group with an HR (95% CI) of 0.97 (0.90, 1.06; P = 0.52; Fig 5). Notably, we observed that race was the source of heterogeneity in the CRPC and M1 metastasis subgroups according to sensitivity analysis. Because the age and patient proportion of the 8 to 10 GS score differed between the Asian and non-Asian baselines, we conducted further analysis to determine whether race was responsible for the subgroup results. Surprisingly, we did not observe any significant difference in the subgroup of patients over and under 68.6-years-old; over 62% of patients were in 8 to 10 GS group, and under 62% were in 8 to 10 GS group with HRs (95% CI) of 0.90 (0.68, 1.20; P = 0.47), 0.97 (0.90, 1.06; P = 0.54), 0.98 (0.88, 1.09; P = 0.7), and 0.85 (0.69, 1.04; P = 0.11), respectively (Fig 6). In addition, heterogeneity was low, with I^2^ values of 0%, 0%, 0%, and 32%, respectively. These results further demonstrate that race may be responsible for the significant OS difference compared with that in the control group.

**Table 2:** Baseline of race.

	Race group			
Baseline Variable	Asian	Not Asian	total	*P* value
No. of patients	5.1%	94.9%	8280	/
HSPC	4.5%	95.5%	7034	/
CRPC	8.4%	91.6%	1246	/
M0	0	100%	3753	/
M1	27.1%	72.9%	1565	/
Median age (years)	71.7	66.9	68.6	<0.0001
Median GS of 8–10 (%)	74%	61%	62%	<0.0001

### Effect of ZA on prostate cancer SREs

Five trials (six groups) yielded a pooled OR and 95% CI of 0.65 (0.45, 0.95; P = 0.02) for SREs based on the random-effects model used to analyze medium heterogeneity (I^2^ = 49%; Fig 2). Our results confirmed that treatment with ZA significantly decreased SRE occurrence compared with that in the control group. However, medium heterogeneity prompted us to adopt a sensitivity analysis to assess the stability of the results. We noted that the study by Kamba et al [19] might be the source of heterogeneity. We observed no prominent high risk of bias in the Tomomi Kamba study [19] after evaluating the bias using the Cochrane software review manager. Thus, we believe that the study and subgroup analyses require further exploration.

**Fig 7:**
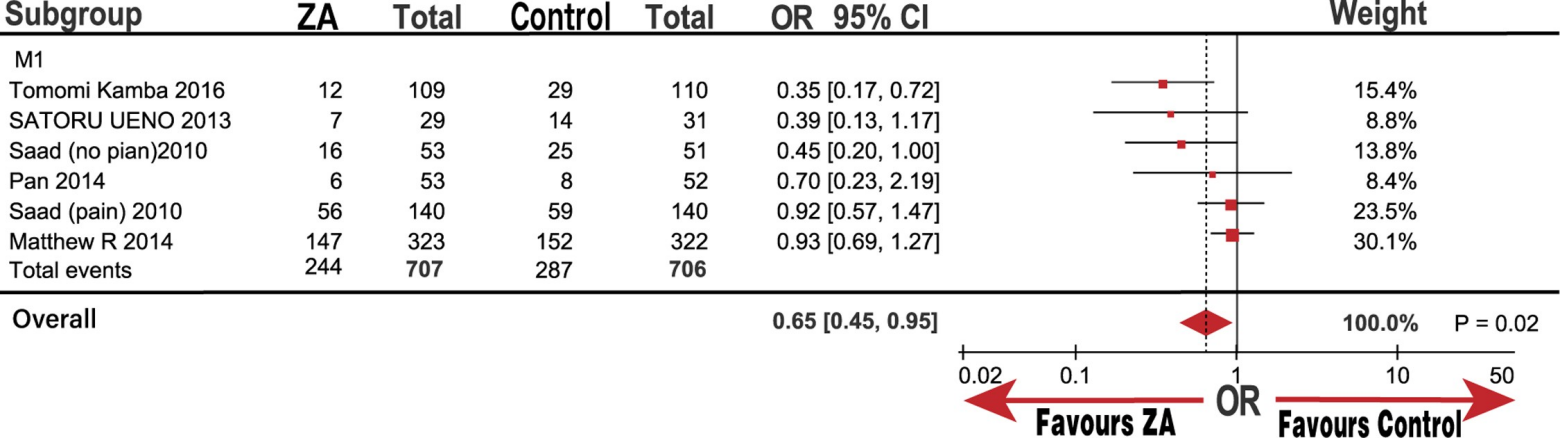
Forest plot odds ratio (OR) of Skeletal related events (SREs) in subgroup of M1.

**Fig 8:**
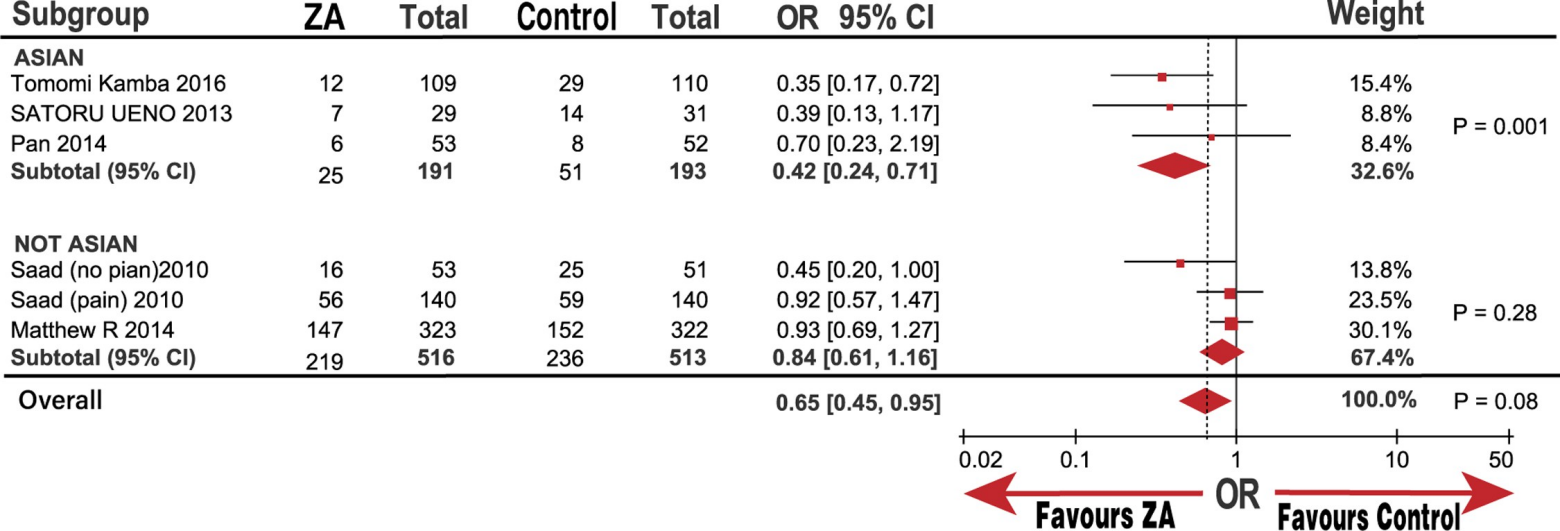
Forest plot odds ratio (OR) of Skeletal related events (SREs) in subgroup of Asian and not Asian.

**Fig 9:**
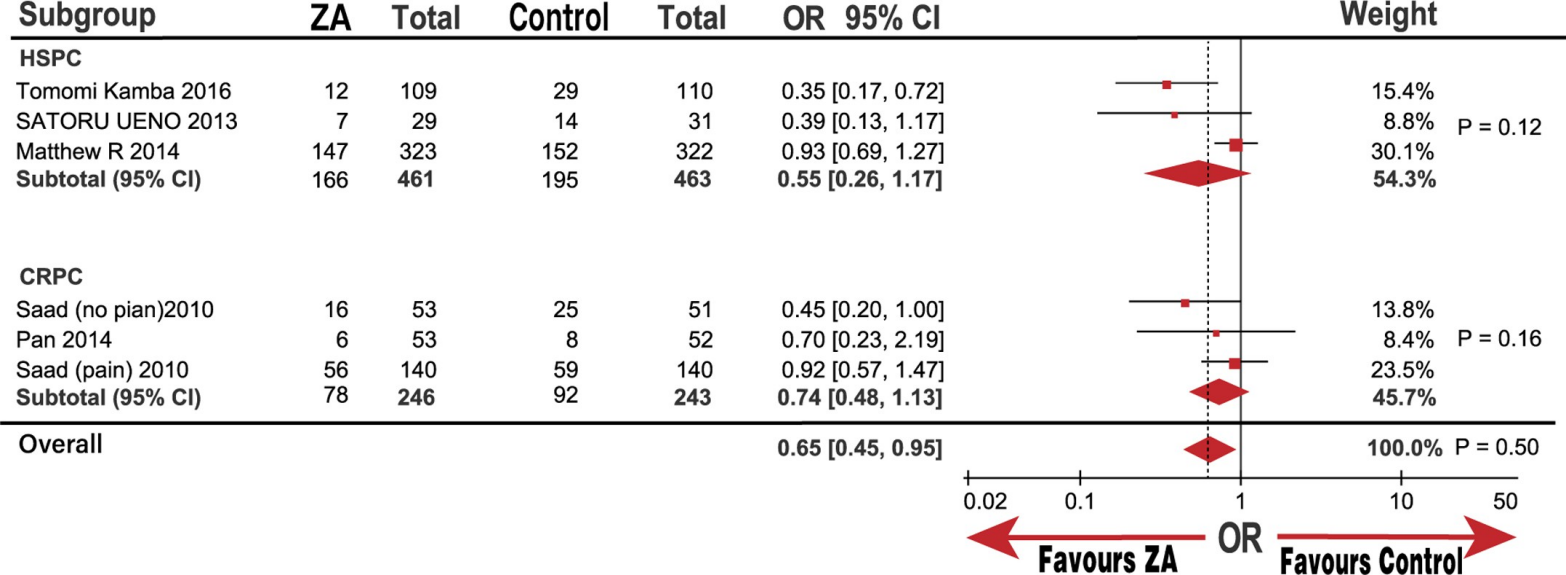
Forest plot odds ratio (OR) of Skeletal related events (SREs) in subgroup of HSPC and CRPC.

**Fig 10:**
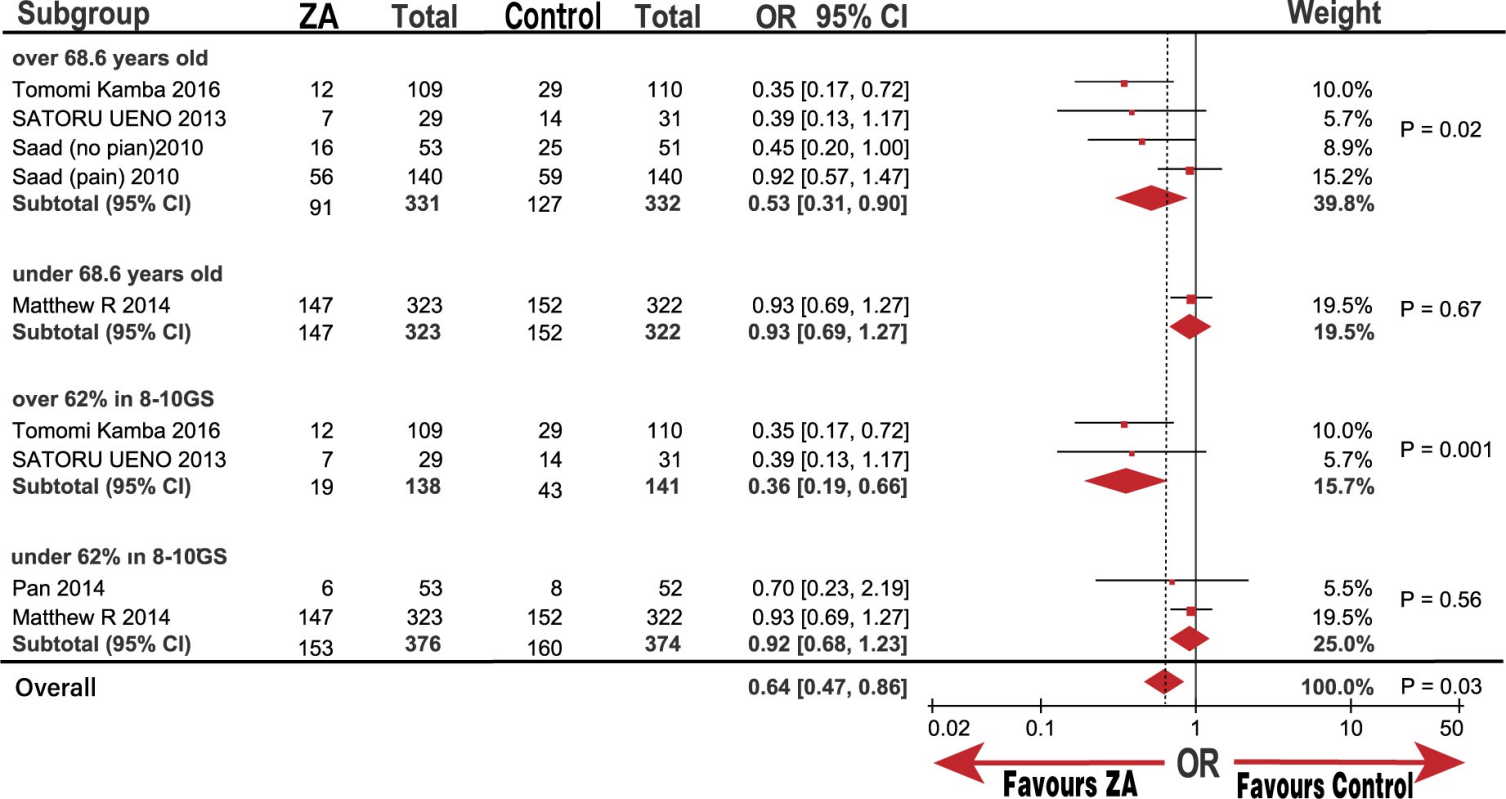
Forest plot odds ratio (OR) of Skeletal related events (SREs) in subgroup of age and GS.

In the subgroup analysis, the SREs significantly decreased in M1 metastasis and the Asian groups with ORs (95%) of 0.65 (0.45, 0.95; P = 0.02) and 0.42 (0.24, 0.71; P = 0.001), respectively. However, there was no significant decline in the CRPC and non-Asian groups, which had ORs (95%) of 0.74 (0.48, 1.13) and 0.84 (0.61, 1.16; Figs 7–9), respectively. Similarly, we determined whether race was responsible for these subgroup results. The groups of patients under 68.6-years-old and over 68.6-years-old were significantly different with an OR (95%) of 0.53 (0.31, 0.90; P = 0.02), but a high heterogeneity (I^2^ = 52%). However, the group of over 62% patients in 8 to 10 GS was significantly different with an OR (95%) of 0.36 (0.19, 0.66; P = 0.001), while the group under 62% of patients with 8 to 10 GS was not significantly different, OR (95%) of 0.92 (0.68, 1.23; P = 0.56). The heterogeneity of both groups was low (I^2^ = 0%) compared with that of the non-Asian group (I^2^ = 30%; Fig 10). The results demonstrate that the percentage of patients with 8 to 10 GS, not their race, was responsible for the significant difference in SREs in the control group. Moreover, sensitivity analysis suggested that the group with 8 to 10 GS, not race, was responsible for the remarkable elevation of heterogeneity in the HSPC group (I^2^ = 74%).

### Effect of ZA on BMD in patients with prostate cancer

**Fig 11:**
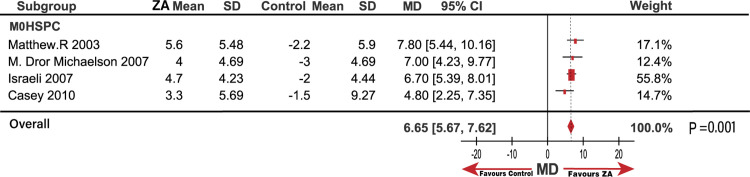
Forest plot with odds ratio (MD) of bone mineral density (BMD) in subgroup of HSPC.

Six trials yielded a pooled MD and 95% CI of 8.08 (5.79, 10.37; P < 0.001) for BMD based on the random-effects model with high heterogeneity (I^2^ = 83%; P < 0.001). The results demonstrated that adding ZA resulted in a remarkable improvement in the BMD value percentage compared to the control group, accompanied by high heterogeneity. The studies by Satoh [27] and Kachnic [25] were suspected to be the source of heterogeneity through sensitivity analysis. A critical review of full-text and evaluation bias conducted by three reviewers according to the Cochrane Review Manager revealed that Satoh [27] recruited M1 metastasis patients, and Kachnic [25] adopted radiotherapy as an additional intervention. However, these two studies had an increased BMD percentage compared to other pooled MD from other studies; thus, we considered these qualified studies. The subgroup of m0HSPC (without Takefumi Satoh [27] and LA Kachnic [25]) analysis also revealed a stable outcome in the increased value improvement of BMD percentage versus the control group, with an MD (95% CI) of 6.65 (5.67, 7.62; I^2^ = 0%; P = 0.001; Fig 11).

## Discussion

This meta-analysis provides evidence that ZA intervention does not significantly improve OS in patients with PCa (HSPC, CRPC, M0, M1) but may improve OS in the Asian population. However, for the OS outcome, the data included a wide range of patients, and heterogeneity must be considered, especially considering the effect of cardiovascular death caused by Androgen Deprivation Therapy (ADT) therapy. Therefore, this conclusion should be conservatively interpreted. SREs significantly decreased in the M1 and Asian groups, and our results also suggested that ZA treatment could improve BMD.

The meta-analysis results of OS were merged by the RCTs, and STAMPEDE was one representative trial that enrolled 2962 men with HSPC. To the best of our knowledge, the role of ZA has only been confirmed in men with mCRPC. In our study, OS for HSPC, mHSPC, and nmCRPC did not benefit from the addition of ZA. This finding was partly consistent with that of Wu [30], who also performed a meta-analysis on bisphosphonates but not specifically zoledronic acid. However, Wu et al. argued that ZA could prolong OS in mHSPC, which is contrary to our findings. We further searched the associated literature and observed no evidence of differences in the efficacy between different types of bisphosphonates [31]. Different types of bisphosphonates did not account for the reversed result. Notably, patients in the HSPC stage had more common competing causes of death, especially death caused by cardiovascular events [32]. This heterogeneity may have caused the controversial finding in Wu’s study. More importantly, many studies have demonstrated a positive association between cardiovascular events or death and CAB [33]. This may be due to the potential heterogeneity between the HSPC and CRPC stages. To offset this difference and obtain a robust outcome, we performed a subgroup analysis of HSPC and CRPC. We observed that the ZA intervention had no significant effect on the OS in the HSPC and CRPC subgroups. These results further demonstrated the pooled outcome of OS. Counterintuitively, in the subgroup analysis by race, we observed that race might impact ZA efficacy. The Asian subgroup exhibited a remarkable improvement in OS, whereas the non-Asian group presented no change. Why did ZA prolong the OS in the Asian group? From the view of tumor biological research, many studies have proven that ZA can suppress the proliferation of PCa cells and other tumor cells [6,34,35]. The antitumor function of ZA implies that ZA may theoretically prolong the OS of patients with PCa. From the perspective of clinical studies, further research is needed to elucidate the relationship between race and PCa. Although pre-clinical studies suggested that ZA had a potential anti-tumor effect, they could not explain its impact on Asian OS. The bias and heterogeneity caused by subgroup analysis should be further elucidated. First, the Asian and non-Asian groups have different treatment strategies, such as ADT alone or CAB. Moreover, these groups both had a proportion of HSPC and CRPC patients. This may lead to non-prostate cancer-specific mortality, such as cardiovascular death, as previously discussed. Although ADT use was not associated with an increased risk of cardiovascular death [36], the significant effect of CAB on cardiovascular death [33] must be considered. Second, the Asian group was defined as patients enrolled in an Asian region, and this may include some patients who live in Asian regions but are not of Asian descent. Although these patients may be a small proportion of all patients, this result should still be interpreted with caution. However, an increasing number of studies have valued race in cancer treatment. A study evaluated survival by race in men with chemotherapy-naive enzalutamide- or abiraterone-treated mCRPC and suggested that black men may have better outcomes than white men [37]. In line with this, another study reported that black men had a statistically significant increase in OS compared to white men [38]. Notably, two other studies argued that black men exhibited poorer survival than white men [39], whereas Asian men had better survival [40]. ZA resulted in a difference in sipuleucel-T immunotherapy, and OS was also significantly different in different races. To date, little is known about the mechanisms by which race affects tumor treatment. We have elucidated that race affects the efficacy of antitumor drugs. Regrettably, our data includes only two trials from China and Japan in the Asian group with a total of 324 participants, whereas five trials from the USA, UK, Germany, and Australia comprise the non-Asian group. Moreover, heterogeneity, such as competing deaths, made the results less robust. Nevertheless, the two Asian trials were reliable RCTs, and the direction for race studies was promising; therefore, we conservatively concluded that the Asian group might have prolonged OS by adding ZA versus the control group.

ZA effectively reduces SRE risk in patients with mCRPC [41]. Our meta-analysis of SREs is consistent with this conclusion, with the subgroup analysis of SREs also indicating that patients with M1 metastasis had a significant decrease in SREs. However, there was no remarkable decrease in the HSPC and CRPC subgroups. These results imply that regardless of the HSPC or CRPC status, patients with bone metastasis can benefit from ZA, but no benefit is gained in patients without bone metastasis. This study supported evidence from clinical observations that ZA use was associated with a decreased SRE risk in patients with a history of SREs; no preventive effects of ZA were observed in patients without a history [13]. However, a review indicated that ZA did not reduce SREs when administered before castration resistance. This conclusion was primarily derived from Smith’s RCT [23], which recruited 645 men with HSPC who were treated with ZA.

Early ZA intervention in mHSPC did not exhibit any benefits. Our meta-analysis, which included RCT by Smith et al., demonstrated opposing results. In this subgroup analysis, we also enrolled two RCTs that recruited men with mHSPC, indicating that our results may be reliable. Surprisingly, SREs also exhibited differences in the GS group. The subgroup with the high portion of GS 8 to 10 revealed a significant decrease in SREs, whereas a low proportion of the 8 to 10 GS group exhibited no statistical difference after ZA treatment. The literature revealed that GS was not directly correlated with a greater risk of developing SREs in a cohort study [42]. However, one cohort held a contradictory opinion that patients were significantly more likely to develop SREs if they had a GS of 8 to 10 [43]. Another study supported this result with the argument that, although there was no statistically significant difference, there was a trend toward an increased risk of SREs among patients with a biopsy GS of 8 to 10 versus those with a GS ≤ 6 [44]. Despite the argument that GS and SREs are associated, further analysis of our data suggested that the two studies both originated from Asia; however, the race group had a higher heterogeneity., Therefore, GS may be responsible for this subgroup’s results. In summary, a high proportion of patients with GS 8 to 10 tended to have a superior cure rate with ZA treatment. If a higher GS predicted a higher risk of SREs, ZA was considered suitable for patients with a high GS.

In terms of BMD outcomes, ZA exhibited favorable preventive effects in HSPC. Notably, Satoh [27] and Kachnic [25], excluded by the subgroup, generated homogeneity. Satoh recruited patients with M1 metastasis, and Kachnic [25] adopted radiotherapy (RT) as an additional intervention, which suggests that patients with M1 and RT treatment exhibited greater improvement in BMD than M0 and no-RT. Notably, BMD benefited from the early use of ZA in patients with M0HSPC in subgroup analysis. Comparing the SREs were significantly decreased in M1 patients; ZA only enhanced BMD, but no SREs reductions were observed in patients with HSPC.

However, there are several inadequacies in this study. First, the heterogeneity was largely owing to the enrollment of a wide range of patients. Thus, we performed a subgroup analysis to partly eliminate heterogeneity; however, this led to other biases. For example, we observed that age and GS of 8 to 10 (%) were significantly different between Asian and non-Asian groups, which may challenge the result of ZA prolonging Asian OS. We subsequently performed a subgroup analysis of age and GS of 8 to 10 (%) to exclude the effect on that result. However, no evidence supports the cut-offs of these subgroups (age and GS of 8 to 10 (%)). We then grouped them by the median, which requires further confirmation. Second, subgroup analysis of Asian and non-Asian patients caused bias in the mixing of different stages of PCa patients and different treatments. Third, in this study, the Asian group referred to patients recruited in Asian countries because some non-Asian patients may have lived in Asian countries and been included, potentially causing heterogeneity. Fourth, due to the lack of sufficient Asian participants, the result of ZA prolonging OS in Asians was not robust. As for the inadequacies above, we interpreted the result with caution and conservation. We hope that more RCTs will be performed to assess the effect of race to update the results of this meta-analysis.

## Conclusions

In summary, ZA supplementation did not significantly improve OS in patients with HSPC, CRPC, M0, or M1. However, our results suggested that the Asian group might have had prolonged OS compared to those in the non-Asian group. Patients with M1 metastasis and the Asian group exhibited a significant decrease in SREs, regardless of HSPC or CRPC status. ZA also prevents bone loss at different stages of PCa.

## Supporting information

S1 Checklist PRISMA 2020 checklist. (DOCX)

S1 Fig Quality assessment of eligible studies. (DOCX)

S2 Fig Funnel plot of OS. (DOCX)

## Abbreviations

ZA: Zoledronic Acid
SOC: standard of care
DOC: Docetaxel
CAB: combined androgen blockade
Sr89: strontium-89
CA: calcium
VD: vitamin D
mCRPC: metastatic castration-resistant prostate cancer
CRPC: Castration Resistant Prostate Cancer
HSPC: Hormone Sensitive Prostate Cancer
mHSPC: metastatic hormone-sensitive prostate cancer
OS: Overall survival
SREs: Skeletal related events
BMD: bone mineral density
RCTs: randomized-control trials
ADT: Androgen Deprivation Therapy
