# Supplementary figures and images for: Do disease status and race affect the efficacy of zoledronic acid in patients with prostate cancer? A systematic review and meta-analysis of randomized control trials
Source: PLoS One. 2022 Sep 22;17(9):e0275176. doi: 10.1371/journal.pone.0275176 (PMC9499269; doi:10.1371/journal.pone.0275176)

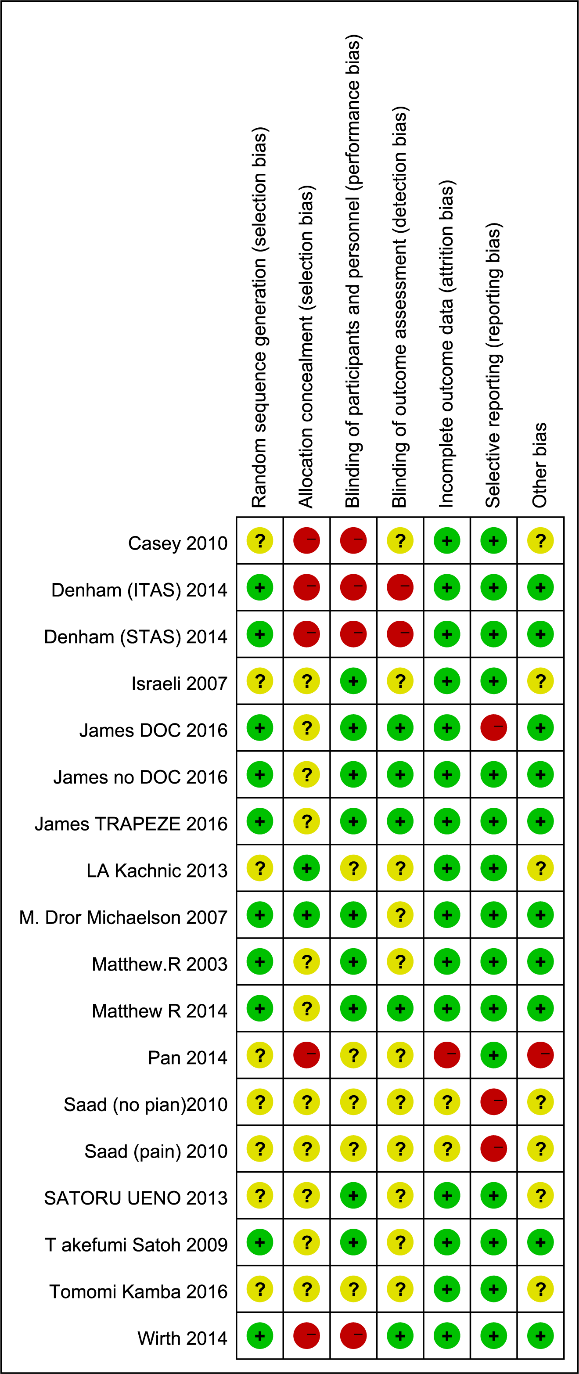


Supplementary Figure S1 Quality assessment of eligible studies

Supplement: S1 Fig — (DOCX) [file pone.0275176.s002.docx]

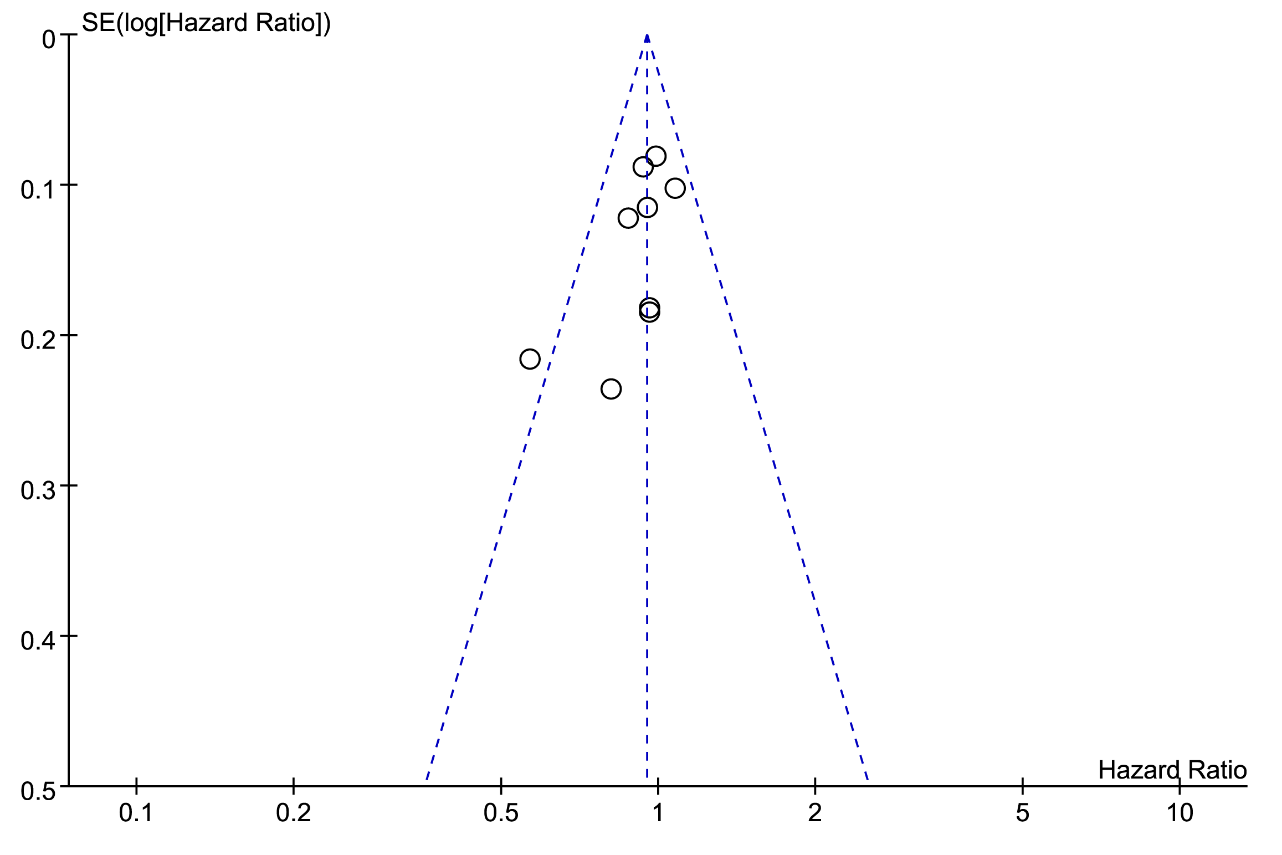


Supplementary Figure S2 Funnel plot of OS

Supplement: S2 Fig — (DOCX) [file pone.0275176.s003.docx]
